# Polygyny does not explain the superior competitive ability of dominant ant associates in the African ant‐plant, *Acacia* (*Vachellia*) *drepanolobium*

**DOI:** 10.1002/ece3.3752

**Published:** 2017-12-27

**Authors:** John H. Boyle, Dino J. Martins, Julianne Pelaez, Paul M. Musili, Staline Kibet, S. Kimani Ndung'u, David Kenfack, Naomi E. Pierce

**Affiliations:** ^1^ Department of Organismic and Evolutionary Biology, and Museum of Comparative Zoology Harvard University Cambridge MA USA; ^2^ Department of Biology The College of William and Mary Williamsburg VA USA; ^3^ Mpala Research Centre Nanyuki Kenya; ^4^ Turkana Basin Institute Stony Brook University Stony Brook NY USA; ^5^ Department of Integrative Biology University of California, Berkeley Berkeley CA USA; ^6^ East African Herbarium, Botany Department National Museums of Kenya Nairobi Kenya; ^7^ Department of Land Resource Management and Agricultural Technology University of Nairobi Nairobi Kenya; ^8^ CTFS‐ForestGEO Smithsonian Tropical Research Institute Washington DC USA

**Keywords:** *Acacia drepanolobium*, ant‐plant, coexistence, colonization, competition, *Crematogaster*, mutualism, polygyny, *Tetraponera*, *Vachellia drepanolobium*

## Abstract

The *Acacia drepanolobium* (also known as *Vachellia drepanolobium*) ant‐plant symbiosis is considered a classic case of species coexistence, in which four species of tree‐defending ants compete for nesting space in a single host tree species. Coexistence in this system has been explained by trade‐offs in the ability of the ant associates to compete with each other for occupied trees versus the ability to colonize unoccupied trees. We seek to understand the proximal reasons for how and why the ant species vary in competitive or colonizing abilities, which are largely unknown. In this study, we use RADseq‐derived SNPs to identify relatedness of workers in colonies to test the hypothesis that competitively dominant ants reach large colony sizes due to polygyny, that is, the presence of multiple egg‐laying queens in a single colony. We find that variation in polygyny is not associated with competitive ability; in fact, the most dominant species, unexpectedly, showed little evidence of polygyny. We also use these markers to investigate variation in mating behavior among the ant species and find that different species vary in the number of males fathering the offspring of each colony. Finally, we show that the nature of polygyny varies between the two commonly polygynous species, *Crematogaster mimosae* and *Tetraponera penzigi*: in *C. mimosae*, queens in the same colony are often related, while this is not the case for *T. penzigi*. These results shed light on factors influencing the evolution of species coexistence in an ant‐plant mutualism, as well as demonstrating the effectiveness of RADseq‐derived SNPs for parentage analysis.

## 1 INTRODUCTION

**Figure 1:**
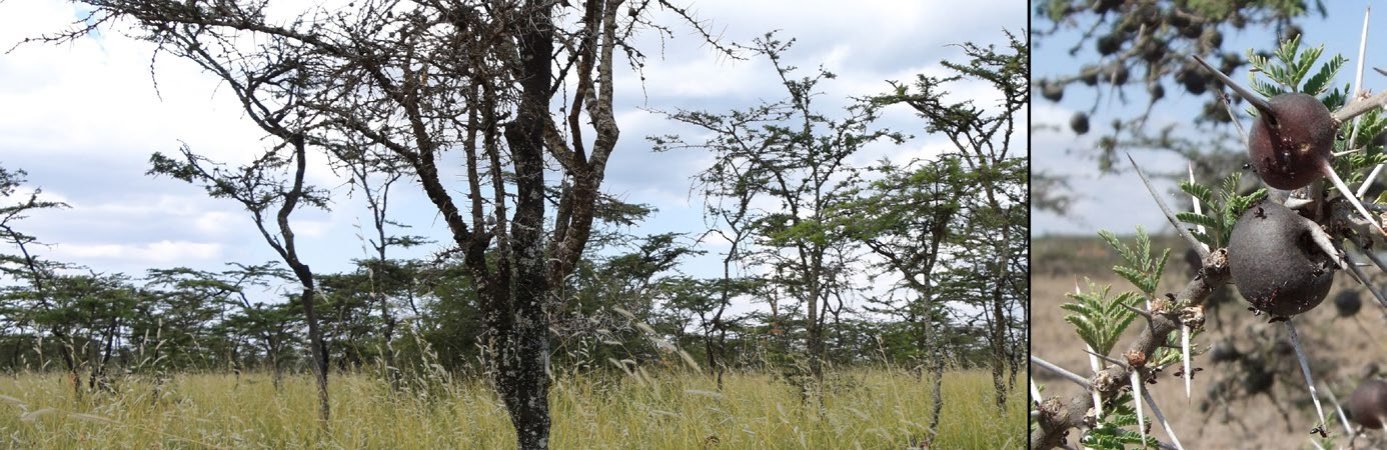
Ants inhabit *Acacia drepanolobium* trees on black cotton soils. On these soils, *A. drepanolobium* may account for 95% or more of trees, as shown on the left‐hand side (Young et al., 1997). Ants live in hollow, swollen thorns and patrol the tree against herbivores (right). Photographs: NEP (left) and JHB (right)

Species coexistence, the question of how different species can coexist while competing for limiting resources, is a central question in ecology (Huston, 1979; Hutchinson, 1961). A classic example of species coexistence is found among four ant species that inhabit the ant‐plant *Acacia* (also referred to as *Vachellia*) *drepanolobium*. The Whistling‐Thorn Acacia, *A. drepanolobium*, is an East African Savannah tree that engages in a defensive mutualism with ants, providing the ants with extra‐floral nectar and housing in hollow, swollen‐thorn “domatia” in return for defense from herbivory (Hocking, 1970; Young, Stubblefield, & Isbell, 1997; Figure 1). At a well‐studied field site in Kenya, four species of ants are commonly hosted by the acacia. Three of them, *Crematogaster mimosae*,* C. nigriceps*, and *Tetraponera penzigi*, are “phytoecious,” or obligate inhabitants of the ant‐plant of *A. drepanolobium*, while the fourth, *C. sjostedti*, is free‐living, nesting under bark and in rotting wood (Stanton, Palmer, & Young, 2002). Almost every mature tree of *A. drepanolobium* is occupied by a single ant colony (although individual colonies can range over a number of adjacent trees), and competition among ants for these trees is intense (Palmer, Young, Stanton, & Wenk, 2000). In light of this, much research has focused on how these four species are able to coexist on a single resource (Palmer, 2003; Palmer et al., 2000; Stanton et al., 2002; Young et al., 1997), in violation of theory suggesting that this situation should be ecologically unstable (Gause, 1934; Hardin, 1960).

The best‐supported hypothesis to explain this coexistence is that a colonization‐competition trade‐off exists among the ant species: ants specialize either in colonizing new resources (i.e., unoccupied trees) or in competing for occupied resources. Observations of naturally occurring transitions from occupancy by one ant species to another have shown a competitive hierarchy among the ants, with *C. sjostedti* as the most dominant, followed by *C. mimosae*,* C. nigriceps,* and *T. penzigi*; this hierarchy has also been supported by experiments in which contests were staged by tying the canopies of neighboring trees together (Palmer et al., 2000). The degree to which a single colony extends over multiple trees (i.e., a form of polydomy, where a single colony has multiple nest chambers spread across several trees) is also correlated with the competitive hierarchy, with *C. sjostedti* occupying the greatest number of adjacent trees, and *T. penzigi* occupying the least (Palmer et al., 2010). Furthermore, more competitive ant species also occupy larger, presumably more valuable trees (Palmer et al., 2000; Young et al., 1997).

Consistent with the competition‐colonization trade‐off hypothesis, a colonization hierarchy runs counter to the competitive hierarchy, allowing species coexistence. Colonization ability has been measured using several metrics: production of foundress queens, recruitment of these foundresses to empty trees, ability of foundresses to win fights with each other over individual domatia on newly colonized trees, speed with which foundresses can produce workers that occupy the rest of the tree, and vulnerability to parasitism (Palmer et al., 2000; Stanton, Palmer, & Young, 2005; Stanton et al., 2002). This research has supported the existence of a colonization hierarchy in which *T. penzigi* is the best colonizer, followed by *C. nigriceps*,* C. mimosae,* and *C. sjostedti*, viz. the reverse order of the competitive hierarchy.

While differences in competitive and colonizing ability among *A. drepanolobium*'s ant associates have been well described, our understanding of the mechanistic basis for these differences is still lagging. The higher competitive ability of dominant species, in particular, seems to be driven by larger worker populations (Palmer, 2004; Ruiz‐Guajardo, Grossenbacher, Grosberg, Palmer, & Stanton, 2017), but the proximate causes of these larger worker populations are unknown. A primary hypothesis proposed is that more competitive species are polygynous, that is, they have multiple queens per colony (Palmer, 2004; Rubin et al., 2013). Collectively, these queens are able to lay more eggs, thereby producing larger worker populations that can outcompete their neighbors. Across ant species, polygyny is associated with large colony sizes and ecological dominance (Boulay, Arnan, Cerdá, & Retana, 2014), including other acacia‐ant mutualisms (Kautz, Pauls, Ballhorn, Lumbsch, & Heil, 2009; McGlynn, 2010), and polygyny is also common among highly competitive invasive ant species (Tsutsui & Suarez, 2003). In the *A. drepanolobium* system, it is unknown whether higher worker populations are the result of polygyny or some other difference(s) among the different ant species. The only research to date on queen number in this system is that of Rubin et al. (2013), who used microsatellite markers to show that *C. mimosae* colonies are commonly polygynous. However, no further work on the remaining three species has been performed to test the hypothesis that queen number underlies competitive ability in these ants.

To learn more about the colony structure of all four common ants in this system, we genotyped multiple same‐colony workers for each of the four species of ant associates using double‐digest restriction‐site associated DNA sequencing (RADseq). This reduced‐representation genomic sequencing method generates DNA sequences of sites near restriction enzyme cut sites, providing a repeatable subset of the genome at a relatively low cost (Peterson et al. 2012). Using hundreds of single‐nucleotide polymorphisms for each of the four ant species, we reconstructed intra‐colony relationships and were able to examine the degree of polyandry and polygyny of the ant species inhabiting *A. drepanolobium*.

## 2 METHODS

### 2.1 Collections

From February to April 2012, ants were collected from *A. drepanolobium* trees tagged in the long‐term monitoring plot of the Center for Tropical Forest Science‐Forest Global Earth Observatories (CTFS‐ForestGEO) at Mpala, Kenya. For each of the four species of ant, about 15 trees were selected, and at least eight workers collected into 95%–100% ethanol. Because only (female) workers were collected, all sequenced individuals were diploid. In June 2015, the sizes of the selected trees were measured using two metrics: height of the tallest part of the canopy, and diameter of the stem at 0.5 m height. The CTFS‐ForestGEO plot is divided into 20 m quadrats; to ensure that we did not sample multiple trees occupied by the same colony, we generally did not sample workers of the same species from trees that occupied the same or neighboring quadrats. When our method of random selection chose trees from the same or neighboring quadrats, we later checked to see whether workers across those colonies shared a parent; we did not find any examples of this. For *C. sjostedti*, which occupies substantially more trees per colony than the other ants (Palmer et al., 2010), we did find two trees sharing some parents that were two quadrats away (about 40 m). At this distance, it is difficult to determine whether these represent one extremely large colony (Palmer et al., 2010 found that *C. sjostedti* colonies occupy on average 22 trees, which represents an area about 12 meters in radius at our site), or two closely related colonies, and so we did not combine the colonies in subsequent analyses.

### 2.2 DNA extraction and sequencing

We extracted DNA from each worker using an AutoGenprep 965 Tissue/ES Cell DNA Extraction Kit, using the Mouse Tail protocol for animal tissue. For this and subsequent steps, we followed the manufacturer's recommended protocols except as described below. Genomic DNA was stored at −20°C before use.

The amount of genomic DNA was then increased by whole genome amplification, using the REPLI‐g mini kit in 15 or 20 μl reactions.

We used the double‐digest restriction‐site associated DNA sequencing (RADseq) protocol of Peterson, Weber, Kay, Fisher, and Hoekstra (2012). We modified their protocol in a number of respects: We started with an (amplified) genomic DNA mass of 150 ng, which we then digested with the restriction enzymes EcoRI‐HF and BfaI under the manufacturer's recommended conditions. Bead cleanups throughout the protocol were performed with a MagNA bead solution described by Rohland and Reich (2012) in place of Agencourt AMPure beads. We added 1.5× volume MagNA beads to the solution to be cleaned, but otherwise followed the same default protocol provided with Agencourt AMPure beads. We used the 48 inline indices for EcoRI described in the Sequences‐S1 spreadsheet in the supplement of Peterson et al. (2012). We chose a range of 264–336 bp for the size selection step, which we performed using 2% ethidium bromide cassettes on a Sage Science Pippin Prep machine. The final PCR was set for 10 cycles.

These libraries were then sequenced in 100 bp, single‐end reads on four lanes of an Illumina HiSeq 2000 and six lanes of a HiSeq 2500 at the Harvard University Bauer Core Facility. The bulk of the libraries for *C. sjostedti*,* C. mimosae*, and *C. nigriceps*, along with a few libraries for *T. penzigi*, ran on six lanes of the HiSeq 2500. Most of the libraries for *T. penzigi*, along with a small number of libraries from the other species, were run on four lanes of the HiSeq 2000.

### 2.3 DNA sequence alignment and base calling

To demultiplex the Illumina libraries, as well as to align reads across worker ants and call single nucleotide polymorphisms (SNPs), we used the program Stacks version 1.21, using the default parameters for this and all software analyses except as described below (Catchen, Amores, Hohenlohe, Cresko, & Postlethwait, 2011; Catchen, Hohenlohe, Bassham, Amores, & Cresko, 2013). Reads were demultiplexed using the *process_radtags* function of stacks, rescuing barcodes and RAD‐tags, and disabling checking if the RAD site was intact.

We quality filtered reads using the FASTX‐Toolkit version 0.0.13 (http://hannonlab.cshl.edu/fastx_toolkit/). For each read, the first seven base pairs, including the EcoRI‐HF restriction site and two often‐low‐quality bases, were removed using the *fastx_trimmer* tool, because the low sequence diversity in this region produced low quality scores. The trimmed reads were then quality filtered using the *fastq_quality_filter* tool, removing any reads with a quality score of less than 25 at more than 2% of bases.

We then aligned all reads for all workers within each species using the *denovo_map.pl* script of Stacks, allowing five mismatches between loci when processing a single individual, and five mismatches when building the catalog. In addition, we explored several different values for these parameters and found that the above combination produced the most SNPs, without substantial increases in heterozygosity that could indicate that different loci were being inappropriately combined (see SI for details). To build the final matrix of SNPs, we culled individuals for which sequencing had failed or had produced too low coverage to be useful (i.e., had <20% SNP coverage when run through a preliminary *populations* run with ‐*r* = 0.5), leaving, on average, about six worker libraries per tree. We called SNPs using the *populations* program of Stacks. A SNP was only processed for the ants from a single tree if it was present in at least *r* of the individuals in the species (*r* cutoffs were set to produce around 500 SNPs total per species: for *C. sjostedti*: 0.5, *C. mimosae*: 0.5, *C. nigriceps*: 0.75, *T. penzigi*: 0.5). We also used the –max_obs_het flag to filter out all SNPs with an observed heterozygosity >0.5, and the –min_maf flag to filter out all SNPs with a minor allele frequency less than 0.02. Heterozygosity was calculated using the adegenet package version 2.0.1 (Jombart, 2008; Jombart & Ahmed, 2011) in R version 3.2.3 (R Core Team 2015).

### 2.4 Relatedness of ants within and between trees

We then compared the average relatedness of each worker ant to every other ant of the same species. We compared the relatedness values across species, for both (i) workers from the same tree (within‐tree) and (ii) workers from different trees (between‐tree). We determined relatedness values using the method of Lynch and Ritland (1999) implemented in the R package *related* (Pew, Muir, Wang, & Frasier, 2015). We chose the Lynch and Ritland method because in simulations it performed better than four other methods of calculating relatedness also implemented in *related*: those of Queller and Goodnight (1989), Li, Weeks, and Chakravarti (1993), Ritland (1996), and Wang (2000). Using Family‐Sim version 1.0 (https://github.com/timothyfrasier/C_software/blob/master/FAM-SIM_v1.0_LINUX_1.tar.gz), the number of alleles recovered for each species, and the allele frequencies observed in our data, for each species we simulated 100 pairs of each of the following relationships between diplodiploid individuals: parent–offspring, full‐sib, half‐sib, and unrelated. For simulated data sets corresponding to the three species of *Crematogaster*, the Lynch and Ritland method had the highest correlation between the true relatedness (0.5, 0.5, 0.25, and 0, respectively) and the relatedness recovered using that method in *related*; while in the case of *T. penzigi*, the Lynch and Ritland method had the second‐highest correlation (*r *=* *.925), after the Queller and Goodnight method (*r *=* *.930). Correlations are shown in the Data S2. For within‐tree comparisons, only relatedness values between workers from the same tree were averaged together (across all workers from the same tree); we then took the mean of these (across all trees of a single ant species) to find the average within‐tree relatedness for each species. For between‐tree comparisons, only relatedness values between workers from different trees were averaged together (across all workers from the same pair of trees); we then took the mean of these to find the average between‐tree relatedness for each species. For *C. nigriceps* and *T. penzigi*, our data set included the genotype of a queen that we happened to collect, one for each species. These two reproductive females were identified as queens by their lack of wings and physogastric abdomens. As the COLONY analysis revealed that the *C. nigriceps* queen was mother to the workers, that individual’s genotypes were excluded from the relatedness analysis; however, the *T. penzigi* queen was sister to the workers collected along with her and was therefore included here.

### 2.5 Within‐tree relationships: polygyny and polyandry

To determine the relationship between individuals collected from the same tree, we used the program COLONY version 2.0.6.3 (Jones & Wang, 2010). COLONY estimates a number of paternal and maternal genotypes, assigning each reconstructed father or mother as a parent of one or more of the observed, genotyped individuals. We ran COLONY under the default run parameters, except for changing the mating system to polygamy for both males and females and the ploidy to haplodiploidy. We set each locus as codominant, with the allele frequency as “unknown,” with an allelic dropout rate of 0.0001 and an additional error rate of 0.0025. The genotypes for two queens, one each for *C. nigriceps* and *T. penzigi*, were included in the offspring genotypes with the worker genotypes. They were also given to COLONY as possible maternal genotypes (alongside any inferred maternal genotypes), with a prior probability of their being the mother of any one of the offspring set at .5 divided by the number of trees in the data set. We used the most likely sibship configuration output to calculate a Polygyny Index and Polyandry Index for each species. We defined the Polygyny and Polyandry Indices as the number of different mothers (i.e., queens) and fathers, respectively, that were estimated to give rise to the workers within each tree. The number of queens and males recovered from the sampled workers is likely to underestimate the total genetic diversity of all workers in a given colony due to the relatively low proportion of workers sampled per colony, but these indices nevertheless allow for the unambiguous comparison of relative degrees of polygyny or polyandry among the different ant species. Furthermore, for each estimated queen, we also looked for multiple mating by recording the number of different males estimated to be the father(s) of her worker offspring. For this purpose, only data from those queens that had at least four offspring among the genotyped workers were used. Finally, for each colony, we calculated a metric of queen dominance by calculating the proportion of the genotyped workers that were daughters of the queen with the most offspring in that colony; we did this for the male that sired the most worker offspring as well.

### 2.6 Maternal relatedness

We recovered the estimated maternal genotypes from COLONY runs, using the same parameters as above, but excluding the possibility that workers from different trees shared a parent. Genotypes for any allele to which COLONY assigned a <.90 probability were recorded as missing data. To determine whether the mothers reconstructed from each tree were related to each other, we ran these putative maternal genotypes in COLONY again, using the same parameters as above, and recorded whether each pair of estimated mothers were full‐siblings, half‐siblings, or unrelated.

### 2.7 Statistical analyses

If colonies with more queens are better able to compete for large and valuable trees, this could produce a correlation between colony structure and tree size. We looked for this by testing whether tree size influenced within‐tree relatedness, Polygyny Index, Polyandry Index, and males per queen, using the Pearson correlation test when the data were approximately normal (or could be transformed to normality: male mates per queen data were square‐root transformed), and Spearman's rank correlation otherwise. We also took into account tree size and ant species using the lm function of R to perform an analysis of covariance (ANCOVA), with height or diameter as the covariate. For each test, there was no significant interaction between ant species and tree size, so we did not include an interaction term in the results presented below. Tree size was measured in two ways, height and diameter at 0.5 m above ground; we present only the results for height here, as height and diameter were strongly correlated and the results of the tests qualitatively identical. The results of tests on tree diameter may be found in Data S3 of the Supporting Information.

As we found no significant effect of tree size on any of these factors, we did not include tree size as a covariate in our final analyses. Comparisons among ant species for between‐tree relatedness, within‐tree relatedness, Polyandry Index, Polygyny Index, and males per queen were performed using analysis of variance (ANOVA) when the data were relatively normally distributed, or Kruskal–Wallis tests (KWT) otherwise. Post hoc comparisons were carried out using Tukey's honest significant difference test (Tukey's HSD) for ANOVA and the Nemenyi post hoc test for KWT.

For between‐tree relatedness, we compared each species’ distribution to zero using Student's one‐tailed *t* test.

For maternal relatedness, we compared the proportion of sibling queens within nests to the proportion of sibling queens between nests using Fisher's exact test.

Statistics were carried out in R. The Nemenyi post hoc tests were performed using the *PMCMR* package (Pohlert, 2014).

## 3 RESULTS

### 3.1 Sequencing and base calling

After DNA sequence filtering, alignment, and SNP calling, we produced genotypes for 300–750 SNPs for each species (Table 1).

**Table 1:** Results of RADseq genotyping: total reads, SNPs per worker ant, total SNPs, and average observed heterozygosity across all SNPs

Species	Trees (colonies)	Workers/tree	Reads/worker	SNPs	Matrix completeness (%)	*H*_obs_
*Crematogaster sjostedti*	16	5.6	356,000	746	56	0.10
*Crematogaster mimosae*	14	6.1	370,000	669	59	0.13
*Crematogaster nigriceps*	18	6.6	420,000	764	84	0.18
*Tetraponera penzigi*	13	5.8	428,000	309	58	0.11

Matrix completeness represents the proportion of loci across all individuals for which a genotype was determined. *H*_obs_, or heterozygosity, is the proportion of individuals that have two different alleles for a given locus, averaged across all loci.

### 3.2 Relatedness of ants within and between trees

**Figure 2:**
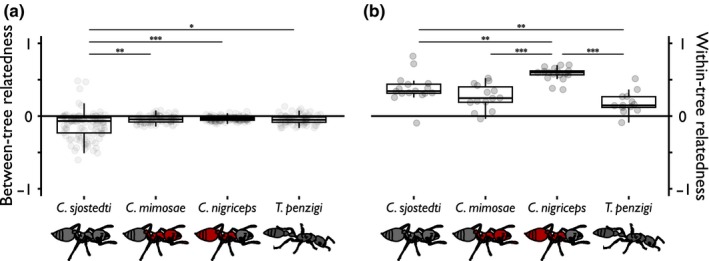
Average relatedness of workers between trees is close to zero, but relatedness within trees is high, and differs among ant species. (a) shows relatedness between ants on different trees (between‐tree comparisons); (b) shows relatedness between ants on the same tree (within‐tree comparisons). The species are arranged left to right in order from most to least competitively dominant. Boxplots show the median and inter‐quartile range for each species. Dots underlying each boxplot show the average relatedness between each pair of trees (a) or within each tree (b); they are jittered horizontally better to show their distribution. Lines above the boxplots denote significant differences between species as follows: *p <* *.05; **p <* *.01; ***p <* *.001. In (a), although the distributions are significantly different among the species, none are significantly greater than zero

The average relatedness of ants within and between trees for each species is shown in Table 2 and Figure 2a. The average relatedness between trees was not significantly greater than zero for any of the ant species (Student's *t* test, *p *>* *.05 for all). There were significant differences in the distributions of relatedness among the four species (KWT, *p *<* *.001; distributions and the results of the Nemenyi post hoc tests are shown in Figure 2a).

**Table 2:** Relatedness of workers within and between trees in the CTFS‐ForestGEO plot at Mpala

Species	Trees sampled	Between‐tree relatedness	Within‐tree relatedness	Polygyny Index	Queen dominance	Polyandry Index	Male dominance	Males per queen
*Crematogaster sjostedti*	16	−0.12 ± 0.02	0.38 ± 0.05	1.4 ± 0.1	0.87 ± 0.05	4.1 ± 0.5	0.46 ± 0.08	3.7 ± 0.5 (*n* = 14)
*Crematogaster mimosae*	14	−0.04 ± 0.01	0.26 ± 0.05	2.9 ± 0.4	0.68 ± 0.07	4.6 ± 0.4	0.35 ± 0.04	3.7 ± 0.3 (*n* = 9)
*Crematogaster nigriceps*	18	−0.04 ± 0.00	0.58 ± 0.02	1.3 ± 0.2	0.97 ± 0.02	2.7 ± 0.3	0.72 ± 0.05	2.4 ± 0.3 (*n* = 18)
*Tetraponera penzigi*	13	−0.05 ± 0.01	0.18 ± 0.04	3.5 ± 0.4	0.54 ± 0.06	4.6 ± 0.4	0.38 ± 0.04	3.0 ± 0.4 (*n* = 4)

Values shown are means ± *SE*.

To calculate the Number of males with which each queen mated, we only considered those queens with at least four offspring among the workers; the number of these queens is given after the number of males per queen in parentheses. Queen dominance is the proportion of genotyped workers who are offspring of the queen with the greatest number of offspring among the genotyped workers. Male dominance is analogous, but for the male with the most offspring. The Polygyny Index is a relative measure that refers to the number of queens per tree as estimated using the data specified in Table 1. The Polyandry Index is similarly a relative measure of the number of males per mated queen estimated from the workers sequenced from each tree.

Tree height did not correlate with within‐tree relatedness either across all ant species (Pearson's correlation test, *p *=* *.8) nor within species (ANCOVA, *p *=* *.7). However, the average within‐tree relatedness did vary among species (ANOVA, *p *<* *.001), as shown in Table 2 and Figure 2b. Post hoc comparisons show that *C. nigriceps* had higher within‐tree relatedness than the other three species, and that *C. sjostedti* had a higher within‐tree relatedness than *T. penzigi* (Tukey's HSD test, *p *<* *.01); this pattern corresponds to the lower polygyny indices of *C. nigriceps* and *C. sjostedti* colonies, discussed below.

### 3.3 Within‐tree relationships: polygyny and polyandry

Across all four ant species, we found no overall correlation between the Polygyny Index and the height of that tree (Spearman's rank correlation, *p *=* *.4), nor did we find evidence for a correlation between Polygyny Index and height within ant species (ANCOVA, *p *=* *.8).

**Figure 3:**
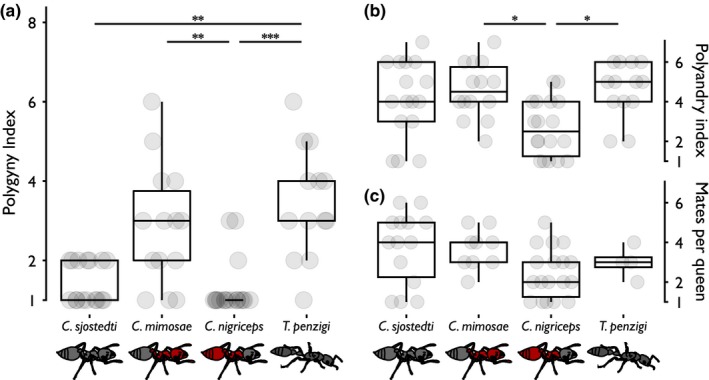
Ant species vary in Polygyny Index, Polyandry Index, and number of male mates per queen. (a) The number of queen genotypes recovered from each tree; (b) the number of male genotypes recovered from each tree; (c) the number of male genotypes recovered from the offspring of each recovered queen (including only those recovered queens with a minimum of four offspring sampled in our data set). The species are arranged left to right in order from most to least competitively dominant. Boxes show median and inter‐quartile ranges. Dots behind each plot show the number of genotypes recovered from each tree (from each queen for c); the values are jittered slightly to help display the data. Lines above the boxplots denote significant differences between species as follows: *p <* *.05; **p <* *.01; ***p <* *.001

However, we did find a strong effect of ant species on degree of polygyny (KWT, *p *<* *.001). *Tetraponera penzigi* and *C. mimosae* typically had higher Polygyny Indices, while *C. sjostedti* and *C. nigriceps* both had Polygyny Indices close to 1 (Table 2, Figure 3a). Post hoc tests found significant differences between *C. mimosae* and *T. penzigi* on the one hand, and *C. nigriceps* on the other, as well as between *T. penzigi* and *C. sjostedti* (Nemenyi tests, *p *<* *.05). In both *C. mimosae* and *T. penzigi*, despite averaging multiple queens per colony, a single queen appeared to be the mother of a disproportionate number of offspring, accounting for 68% and 54%, on average, of the genotyped workers. Finally, for the two queens recovered during sampling, the *C. nigriceps* queen was recovered as the mother of the workers from that tree, while the *T. penzigi* queen was recovered as sister to her colony mates, rather than as their mother.

Across all species, we found no significant correlation between the Polyandry Index and the height of that tree (Spearman's Rank Correlation, *p *=* *.6), or any correlations for one or more individual species (ANCOVA, *p *=* *.6).

The Polyandry Index differed among ant species (KWT, *p *<* *.01). For *C. sjostedti*,* C. mimosae*, and *T. penzigi*, the workers collected from each tree were fathered by about 5–6 males, whereas for *C. nigriceps,* workers were fathered by about half that many males (Table 2, Figure 3b). Post hoc tests found significant differences only between *C. nigriceps* on the one hand and *C. sjostedti* and *C. mimosae* on the other (Nemenyi tests, *p *<* *.05).

We found no significant correlation between the number of males with which each queen had mated (including only queens that were the mothers of at least four genotyped progeny) and the height of the tree occupied by those ants (Pearson's correlation test, *p *=* *.5). We also did not find evidence for a correlation between queen polyandry and tree size for any single ant species (ANCOVA, *p *=* *.9).

The queens of different species showed no significant differences in their number of matings (ANOVA test, *p *=* *.06). Queens of *C. sjostedti* and *C. mimosae* averaged 3–4 mates per queen, while *C. nigriceps* and *T. penzigi* were around 2–3 (Table 2, Figure 3c). These (nonsignificant) differences may be due to chance, or to a reduced sample size, as some queens were excluded from this analysis because they had fewer than four offspring among the workers.

### 3.4 Maternal relatedness

For *C. sjostedti* and *C. nigriceps*, only a few of the inferred queens came from the same tree; however, some of these queens were related to each other (Table 3). *Crematogaster mimosae* had multiple inferred queens per tree. These were also commonly siblings, at a higher rate than that found between queens from different trees (Fisher's exact test, *p *<* *.05). However, *T. penzigi* showed a different pattern: despite having multiple inferred queens per tree, these queens were very rarely siblings, and the rate was not significantly different from the rate of sibship between queens from different trees (*p *=* *.3). The queen we collected from a *T. penzigi* colony was determined by COLONY to be a sister to the workers in that tree.

**Table 3:** Relatedness of inferred queens

Species	Number of trees	Number of inferred queens	SNPs	Matrix completeness (%)	Proportion of related queens from the same tree	Proportion of related queens from different trees
*Crematogaster sjostedti*	16	21	746	100	0.20 (1/5)	0.04 (8/205)
*Crematogaster mimosae*	14	35	669	100	0.20 (8/39)	0.09 (52/556)
*Crematogaster nigriceps*	18	22	764	100	0.14 (1/7)	0.01 (2/246)
*Tetraponera penzigi*	13	39	309	100	0.09 (4/44)	0.06 (39/697)

For each proportion, the raw number of related queens and possible comparisons is given in parentheses.

## 4 DISCUSSION

We found no evidence that the genetic structure of colonies—in terms of polygyny, polyandry, or within‐tree relatedness—underlies competitive ability, either within or between species, in the ant associates of *A. drepanolobium*.

Between species, there was no association between polygyny and competitiveness: Our results do not support the hypothesis that the competitive *C. sjostedti* and *C. mimosae* are more polygynous than the less competitive *C. nigriceps* and *T. penzigi* (Palmer, 2004; Stanton et al., 2002). Palmer (2004) observed seven *C. sjostedti* queens in a single colony fragment, but we found little evidence for polygyny in this species and suspect that the observed queens were not yet mated, but were still in the process of budding off from their natal colony. *C. sjostedti* alates and foundress queens are rarely found, and this species is believed to reproduce primarily via colony budding (Stanton et al., 2002).

Our results also show that *T. penzigi* colonies are polygynous. A previous report indicates that this species was monogynous, based on the dissection of colonies (cited in Stanton et al., 2002). We have also observed that small trees containing colonies of *T. penzigi* tend to have only one laying queen, based on the collection of the ants inhabiting about 20 small (averaging 1.0 m high) trees of *T. penzigi* in 2016 (Boyle and Pierce, *unpublished data*). It is possible that the multiple maternities of workers observed a in single colony of *T. penzigi* are partly a relic of the colony founding event, as seedling stems of *A. drepanolobium* (considerably younger than the small trees surveyed for queens above) typically have a foundress queen in every domatium, and these foundresses are disproportionately *T. penzigi* (Stanton et al., 2002). Possibly the victorious foundress and/or her workers eliminate the competing queens, but tolerate their worker offspring, or continue to raise their brood, as do slave‐making ants. This scenario would result in a colony with a single queen, but a worker population with multiple mothers. This would also be consistent with our finding that a disproportionate number of workers are the offspring of a single queen (about half). This hypothesis could be tested by following individual colonies of *T. penzigi* over time, with the expectation that the colony would become more genetically homogenous as the absorbed workers died out and were replaced by the queen's daughters; if the colony remained genetically heterogenous over time, this would favor either multiple queens or the constant absorption of workers from neighboring *T. penzigi* colonies. Whatever the explanation, even if *T. penzigi* colonies are socially monogynous, it remains the case that polygyny does not underlie the competitive hierarchy, as only one of the two more competitive species, *C. mimosae*, showed evidence of polygyny.

The nature of polygyny also appears to differ among the different species of ants. In the polygynous colonies of *C. mimosae*, multiple queens are often related to each other, possibly due to daughter queens remaining in their natal nests. This was suggested by Rubin et al. for *C. mimosae* (2013), and it may also be the case for the rare colonies of *C. sjostedti* and *C. nigriceps* for which we found evidence of polygyny (i.e., a Polygyny Index > 1). These *Crematogaster* colonies also accepted unrelated queens (or at least, accepted the offspring of unrelated queens), although this appeared to be less common in these ant species than in *T. penzigi*. Queens in polygynous colonies of *T. penzigi*, in contrast to the other three species, were rarely related to each other, which is consistent with the possibility that the multiple maternity of *T. penzigi* workers is a relic of competing foundresses.

The Polygyny Index we present here is not an exact estimate of the true genetic diversity within a tree. When the number of queens and/or males is high relative to the number of workers sampled per tree, as for *C. mimosae*, then our estimates are likely to be underestimates. Further work with larger worker sample sizes will be necessary to infer the absolute degrees of polygyny and polyandry. However, to evaluate whether different colony structures underlie competitive ability, we only need to determine whether the competitively dominant species are more polygynous and/or polyandrous than the competitively subordinate species. Our results provide a sufficiently strong estimate of the relative degree of polygyny and/or polyandry among these four ant species that we can rule out this possibility.

Colonies of *C. sjostedti* and *C. mimosae* usually occupy multiple trees (and *C. nigriceps* moderately so; see Palmer et al., 2010), and if workers from different trees are not thoroughly mixed, then sampling from a single tree will only capture a subset of the genetic lineages in a colony. However, there is good reason to believe that the workers do mix well, as Young et al. (1997) noted that *C. sjostedti* and *C. mimosae* ants readily move along the ground between trees, and we have observed this ourselves. Moreover, Palmer created artificial barriers to the flow of workers between different trees of the same colony and found that this reduced competitive ability to the extent that the colonization hierarchy could be reversed (2004). Both observations suggest that workers among trees within a single colony show sufficient movement that sampling from single trees, as we did, is adequate to capture the diversity of the entire colony, even if it is spread over more than one tree.

Our results also do not support the hypothesis that polygyny plays a role in intra‐specific contests. Across the four species, more competitive ants occupy larger, more valuable trees; however, we did not find an association between queen number and tree size, either within or across ant species. However, as single ant colonies can occupy multiple trees, it is still possible that more polygynous colonies spread across a greater number of trees of the same size class than do their less polygynous conspecifics.

Two lines of research arise naturally from the findings we present here. First, if polygyny does not contribute to interspecific (or intraspecific) variation in colony size, what factors do? Some alternative hypotheses, such as differences in how resources are exploited, or how they are allocated to workers versus reproductives, have already been proposed (Palmer, 2004) and could be explicitly tested. Second, the variation we do observe in polygyny merits greater investigation at both proximate and ultimate levels. Our data suggest that both polygynous species may commonly accept unrelated queens and/or their offspring, but that *Crematogaster* spp. colonies may also contain multiple related queens; however, a finer‐grained description of these patterns could also shed light on the consequences of polygyny (and polyandry) in these species. For instance, honeybee colonies with multiple patrilines reproduce more than single‐patriline colonies, possibly because a genetically diverse worker population is able to forage across a wider range of conditions (Mattila & Seeley, 2007). While polygyny and polyandry do not seem to affect colony size, they may still have consequences for the fitness both of the ant colonies in question and their host trees.

Our work demonstrates the utility of RADseq data in determining family structure, especially in nonmodel systems. RADseq has previously been used to identify parent–offspring relationships where the pool of possible parents is known and genotyped (Kess, Gross, Harper, & Boulding, 2016), and to search for related individuals within a population (Hellmann et al., 2016; Kjeldsen et al., 2016). To our knowledge, our study is the first to use RADseq data to determine the number of parents giving rise to a set of (potentially sibling) offspring, and the first to use RADseq data to determine any kind of kinship relationship in a social insect. This information is important in social insects, for which polygyny and polyandry are both of particular relevance in the evolution and maintenance of eusociality (Boomsma, Kronauer, & Pedersen, 2009). This analysis can also answer other questions, such as quantifying extra‐pair paternity. Although RADseq produces single‐nucleotide polymorphisms, which are individually less informative than microsatellite markers, it produces a great many of them, allowing relationships to be resolved with even greater specificity than with microsatellites. For instance, Weinman et al. found that 102 SNPs had power comparable to 15 microsatellites for identifying parentage in a cooperatively breeding bird, and in fact the SNPs performed better when parents were related (2014), as appears to be the case among some of the ant associates of *A. drepanolobium*. RADseq is especially useful in the many systems for which microsatellite markers have not yet been identified, as no pre‐existing genomic information is needed in order to recover RADseq markers (unlike microsatellites, for which loci must be identified and primers designed beforehand, a potentially expensive and time‐consuming process). In our case, RADseq markers allowed us to conclude that polygyny does not drive competition in the ant associates of *A. drepanolobium,* and we should investigate other factors that could promote differences in competitive ability between species, such as distribution of colony biomass among different castes and foraging differences (Palmer, 2004), or differences in relative rates of development, in diet, and/or abilities to form cohesive colonies across multiple trees.

Interactions among *A. drepanolobium*'s ant associates are not uniform across East Africa. For example, Hocking noted that the proportion of trees occupied by particular ants varied widely from site to site: He rarely found *C. sjostedti* in his sites in the southern part of the *A. drepanolobium* range (1970). This ant, however, is the competitively dominant species in the northeastern part of the range of *A. drepanolobium* at the Mpala Research Centre where most of the research on this system has taken place. Similarly, competitively dominant ants in some habitats may be subordinate in others: Palmer found one site with a different soil profile where the competitive hierarchy was partially reversed, with *C. nigriceps* dominant over *C. mimosae* (2004). To understand how and why the competitive hierarchy changes across the range of *A. drepanolobium*, as well as the ecological consequences of these changes, it will be necessary to understand more about factors that promote competitive ability between the different ant species.

## CONFLICT OF INTEREST

None declared.

## DATA ACCESSIBILITY

Sequencing files may be found in the NCBI Sequence Read Archive, BioProject PRJNA414448. Scripts for filtering, SNP calling, etc., R scripts for statistical analyses, and tree size data may be found in dryad, https://doi.org/10.5061/dryad.g0t42

## AUTHOR CONTRIBUTIONS

JHB, DJM, and NP designed the study. JHB, DJM, and JP did the fieldwork to collect the ants. PMM, SK, SKN, and DK provided data on the trees. JHB generated and analyzed genotype data. JHB and NP wrote the manuscript with the input of all co‐authors.
