# Supplementary material for: Polygyny does not explain the superior competitive ability of dominant ant associates in the African ant‐plant, Acacia (Vachellia) drepanolobium
Source: Ecol Evol. 2017 Dec 27;8(3):1441–50. doi: 10.1002/ece3.3752 (PMC5792509; doi:10.1002/ece3.3752)
Supplement: Supplementary file 1 [file ECE3-8-1441-s001.docx]

**Supporting information**

**Data S1:** Outputs from Stacks under a variety of parameters.

When building the Stacks catalogs of loci, we allowed two parameters to vary: the number of mismatches allowed between loci when processing an individual (-M parameter), and the number of mismatches allowed between loci when building the catalog (-n parameter). To compare the outcomes, we then used the same set of individuals and run parameters described in the main body of the paper to call SNPs from each catalog. We then calculated heterozygosity using the adegenet package version 2.0.1 (Jombart 2008, Jombart and Ahmed 2011) in R version 3.2.3 (R Core Team 2015).

| Species | -M | -n | SNPs | Missing data | Heterozygosity |
| --- | --- | --- | --- | --- | --- |
| *C. sjostedti* | 1 | 1 | 156 | 0.43 | 0.10 |
| *C. sjostedti* | 2 | 1 | 190 | 0.43 | 0.12 |
| *C. sjostedti* | 2 | 2 | 507 | 0.44 | 0.10 |
| *C. sjostedti* | 2 | 5 | 534 | 0.44 | 0.08 |
| *C. sjostedti* | 5 | 1 | 245 | 0.43 | 0.13 |
| *C. sjostedti* | 5 | 5 | 746 | 0.44 | 0.10 |
| *C. mimosae* | 1 | 1 | 201 | 0.39 | 0.12 |
| *C. mimosae* | 2 | 1 | 257 | 0.39 | 0.13 |
| *C. mimosae* | 2 | 2 | 546 | 0.41 | 0.12 |
| *C. mimosae* | 2 | 5 | 611 | 0.41 | 0.11 |
| *C. mimosae* | 5 | 1 | 295 | 0.40 | 0.14 |
| *C. mimosae* | 5 | 5 | 669 | 0.41 | 0.13 |
| *C. nigriceps* | 1 | 1 | 427 | 0.16 | 0.18 |
| *C. nigriceps* | 2 | 1 | 515 | 0.15 | 0.19 |
| *C. nigriceps* | 2 | 2 | 688 | 0.16 | 0.18 |
| *C. nigriceps* | 2 | 5 | 678 | 0.16 | 0.18 |
| *C. nigriceps* | 5 | 1 | 566 | 0.15 | 0.18 |
| *C. nigriceps* | 5 | 5 | 764 | 0.16 | 0.18 |
| *T. penzigi* | 1 | 1 | 190 | 0.40 | 0.09 |
| *T. penzigi* | 2 | 1 | 151 | 0.41 | 0.13 |
| *T. penzigi* | 2 | 2 | 262 | 0.41 | 0.10 |
| *T. penzigi* | 2 | 5 | 323 | 0.42 | 0.07 |
| *T. penzigi* | 5 | 1 | 242 | 0.42 | 0.14 |
| *T. penzigi* | 5 | 5 | 309 | 0.42 | 0.11 |

**Data S2** The Lynch and Ritland method of calculating relatedness performed best in simulated data sets.

| Species | Queller and Goodnight 1989 | Li *et al.* 1993 | Ritland 1996 | Lynch and Ritland 1999 | Wang 2000 |
| --- | --- | --- | --- | --- | --- |
| *C. sjostedti* | 0.9720 | 0.9665 | 0.9526 | 0.9724 | 0.9656 |
| *C. mimosae* | 0.9730 | 0.9719 | 0.9531 | 0.9751 | 0.9711 |
| *C. nigriceps* | 0.9788 | 0.9752 | 0.9664 | 0.9792 | 0.9754 |
| *T. penzigi* | 0.9299 | 0.9237 | 0.8633 | 0.9249 | 0.9204 |

The *r* value given for in each cell is the correlation between the true relatedness value and the relatedness value calculated using each method from simulated data.

**Data S3** Stem diameter produces the same results as tree height when used as a measure of tree size.

When trees had multiple stems at 0.5 meters each greater than 1 cm in diameter, we calculated the final diameter, *d*, as *d*^2^ = Σ*d_i_*^2^, where *d_i_* is the diameter of each individual stem at 0.5 meters.

Tree height and stem diameter were closely correlated (r^2^ = 0.74, see the figure below), and using stem diameter instead of height did not make a difference for the result of any of the tests performed in this paper (see the table below: a “yes” in the rightmost column indicates that the tests using height and the tests using diameter were both significant or both insignificant at a significance threshold of *p* < 0.05.


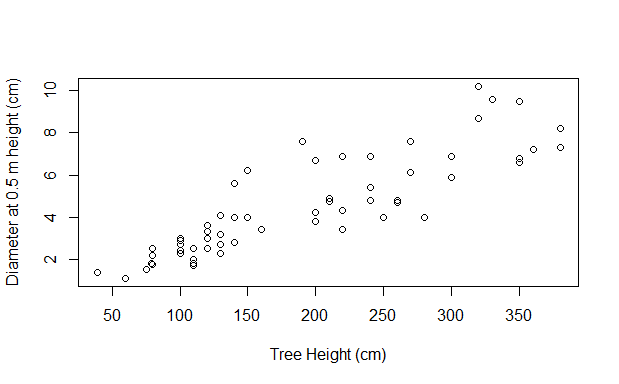


| Analysis | *p*-value | Same as height? |
| --- | --- | --- |
| Pearson correlation test between diameter and average intra-colony relatedness | 0.6 | yes |
| ANCOVA of diameter and ant species on intra-colony relatedness | 0.8 | yes |
| Spearman’s rank correlation between diameter and queen number | 0.2 | yes |
| ANCOVA of diameter and ant species on queen number | 0.3 | yes |
| Spearman’s rank correlation between diameter and male number | 0.9 | yes |
| ANCOVA of diameter and ant species on male number | 0.5 | yes |
| Pearson correlation test between diameter and queen mate number | 0.2 | yes |
| ANCOVA of diameter and ant species on queen mate number | 0.4 | yes |
